# Development and Effect of an Interactive Simulated Education Program for Psychological First Aid: A Randomized Controlled Trial

**DOI:** 10.1155/2024/8806047

**Published:** 2024-08-22

**Authors:** Eun-Joo Choi, Yun-Jung Choi

**Affiliations:** ^1^ Kyung-In Women's University Department of Nursing, Incheon, Republic of Korea; ^2^ Chung-Ang University Red Cross College of Nursing, Seoul, Republic of Korea

## Abstract

**Background:**

Considering the importance of psychological first aid, which is the first priority when a disaster occurs, developing a web-based simulation training program for nurses and confirming its effectiveness is necessary.

**Aim:**

This study aimed to develop an interactive simulated education program as a psychological first aid program for nurses and verify its effectiveness. *Participants*. Nurses working in hospitals and the community who had not participated in psychological first aid training in the last year were recruited.

**Methods:**

A web-based interactive simulated educational program for psychological first aid was developed. To verify its effectiveness, a randomized controlled trial design was used. The experimental group participated in a web-based educational program, while the control group was provided self-learning data in the form of e-books. The program's effects on disaster response core competencies, problem-solving abilities, and self-leadership capacity were measured. We used descriptive statistics to analyze the general characteristics, and independent *t*-tests were used to analyze the differences before and after the intervention.

**Results:**

The core competencies for disaster response (*t* = −2.239, *p* < 0.05, Cohen's *d* = 0.59), problem-solving abilities (*t* = −2.753, *p* < 0.01, Cohen's *d* = 0.72), and self-leadership capacity (*t* = −2.073, *p* < 0.05, Cohen's *d* = 0.54) showed a statistically significant difference between groups.

**Conclusions:**

The web-based simulation education program for psychological first aid training developed in this study effectively enhanced nurses' ability to respond to disasters and improved their problem-solving abilities and self-leadership capacity. Thus, nurses can use the educational program as a tool to learn psychological first aid. This trial is registered with KCT0008965.

## 1. Introduction

Earthquakes are natural disasters with an immense capacity for destruction that cause large-scale casualties and enormous economic losses over a short period of time. In addition to social and economic damage, they can cause psychological problems among victims [1]; residents of earthquake-damaged areas experience long-term anxiety, depression, post-traumatic stress, and suicidal thoughts [2]. They may find it difficult to return to daily life and experience post-traumatic stress disorder if such psychological problems are overlooked [3].

To overcome this challenge, psychological first aid (PFA) is provided to victims at a disaster site. This refers to the activities conducted immediately after a disaster to help victims with their practical needs and reduce their psychological problems [3, 4]. As an essential activity, PFA is prioritized at disaster sites to meet the psychological needs of disaster victims and prevent post-traumatic stress disorder [5]. To effectively perform PFA, one must be familiar with the characteristics of disasters and psychological problems of victims, in addition to having disaster response capabilities to cope with such situations [6]. However, practitioners deployed at disaster sites experience empathy fatigue, secondary trauma, and exhaustion while performing PFA. Therefore, increasing their ability to care for and manage their needs is crucial [7]. For example, when self-leadership is high, work performance [8] and problem-solving improve [9].

Disasters have characteristics that are difficult to fully reproduce through traditional methods such as role-play or simulation education [10]. However, with varying degrees of realism and fidelity, it is possible to effectively simulate disaster scenarios. As such, providing learners with a sense of realism through web-based simulation education is important [11, 12]. Learning through experience is an important element of simulations; accordingly, Kolb [13] emphasized the importance of experience in learning, explaining that learning is a continuous process that combines experience, cognition, perception, and action.

Online education has seen a significant rise in popularity in recent times, with gradual use of web-based simulation education [14]. Web-based simulations have the advantage of transcending spatial and temporal limitations [15] and allowing for repeated learning and immediate feedback [16].

Web-based virtual practice uses standardized patients rather than graphics to ensure learners experience a higher sense of realism [17]. Considering the importance of PFA, developing a web-based PFA simulation training program can be an effective educational strategy for expanding learners' capabilities. Therefore, this study aimed to develop a web-based interactive PFA simulation training program and verify its effects on the disaster response core competencies, problem-solving, and self-leadership of nurses involved in earthquake relief.

## 2. Methods

### 2.1. Study Design

#### 2.1.1. Development of the Web-Based PFA Simulated Education Program

The program was developed following the five stages of the ADDIE model (Analysis, Design, Development, Implementation, and Evaluation).

In the analysis stage, major educational topics and content were developed based on both domestic and international education materials and practical guidelines on PFA. To identify education needs, interviews were conducted with eight community nurses who had completed PFA training. These interviews revealed the necessity for realistic and highly immersive education, the opportunity for repeated participation, and an online format for the training.

In the design stage, educational objectives and content were structured for each phase, including pre-learning, pre-briefing, simulation, and debriefing.

The process in the development stage comprised three phases. The first phase involved developing the simulation scenarios and quizzes. The researcher created the content through a literature review, preliminary needs assessment, and consultations with one disaster mental health expert, two case managers, and nine earthquake disaster victims in South Korea. The second phase involved producing the simulation videos, wherein standardized patients and nurses acted according to the scenarios. The third phase involved creating multimedia content following a storyboard that was developed using Articulate Storyline 3.

In the implementation stage, the developed content was tested and evaluated. Six experts in system development conducted a heuristic evaluation to identify issues with the system interface [18]. The evaluation scores averaged 4.26 ± 0.22 out of 5 points, and the converted score out of 100 was 85.15 ± 4.47. Additionally, to verify the usability and educational effectiveness of the online program from the user's perspective, user evaluations were conducted with 10 nurses holding at least a master's degree. The educational content was evaluated using a 5-point Likert scale. The usability and usefulness of the content were assessed using a tool for evaluating virtual gaming simulation [19].

The evaluation, usability, and usefulness of the content averaged 4.46 ± 0.46, 4.41 ± 0.43, and 4.45 ± 0.44 out of 5 points, respectively. The converted scores, respectively, were 89.17 ± 9.14, 88.20 ± 8.56, and 89.00 ± 8.76 out of 100 points. Additional feedback included issues such as video playback stopping intermittently, requests to change the position of the navigation buttons, providing information on the sequence of the quiz questions, and reducing the display time of the quiz pop-ups. The results and feedback from experts and user evaluations were incorporated into the revisions, and the final version of the content was based on these inputs.

Lastly, the evaluation stage aimed to confirm the effectiveness of the developed web-based program.

#### 2.1.2. Evaluation of the Web-Based PFA Simulated Education Program

**Figure 1:**
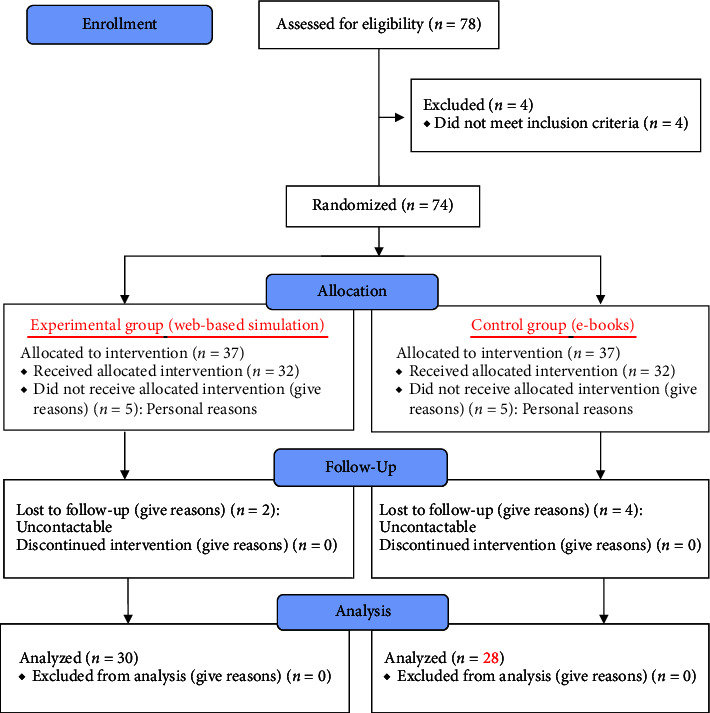
CONSORT diagram.

This study was designed as a single-blind randomized controlled trial to evaluate the effectiveness of the program. The effects of the program were evaluated by a single researcher. The principal researcher generated the random allocation sequence, enrolled participants, and assigned participants to interventions. The guidelines outlined in the CONSORT statement for reporting randomized controlled trials were adhered to. Participants were randomly assigned to either the experimental group, which received the web-based PFA interactive simulated education, or the control group, which received self-learning materials in the form of e-books. To maintain a single-blind design, the group assignments were known only to the researchers to properly administer the interventions, and not to the participants (Figure 1).

### 2.2. Participants

The inclusion criteria for participation included being nurses working in hospitals and the community who had not participated in PFA training in the last year as well as having access to a personal computer and being able to use it without difficulty. The exclusion criteria included nurses who were undergoing treatment for any illnesses or who had difficulty operating the online program. The sample size was calculated using G^∗^power 3.1.9.2. Based on a comparison of two groups (*t*-test) in simulation-based intervention studies [20], the required sample size was determined with a significance level (*⍺*) of 0.05, power (1-*β*) of 0.80, and effect size (d) of 0.8. The results indicated that 52 participants were needed. Considering the potential dropout rate, the sample size was increased to 60, with 30 participants in the experimental group and 30 in the control group. The researcher posted recruitment notices on social network communities of hospitals and organizations in five South Korean cities and explained the purpose and methods of the study to the potential participants. Written consent was obtained from the nurses who agreed to participate in the study.

Initially, 74 participants agreed to participate and completed the pre-survey questionnaire. Serial numbers were assigned in the order of registration to the study participants and the SPSS randomization function was used to determine the experimental and control groups. The participants were not informed to which group they belonged. After completing the pre-survey questionnaire and prior to the intervention, two participants from the experimental group of 37 could not be contacted, and five could not participate owing to personal reasons. In the control group of 37, four participants could not be contacted and five could not participate owing to personal reasons. The final number of participants was 30 in the experimental group and 28 in the control group.

### 2.3. Measurements

To evaluate the effectiveness of the program, we administered the Perceived Competence Scale for Disaster Mental Health Workforce (PCS-DMHW) to the participants and assessed their problem-solving ability and self-leadership capacity both before and after the intervention. The pre-assessment also included one additional section on participants' sociodemographic characteristics, including sex, age, religion, years of work experience, field of work, and history of PFA training.

#### 2.3.1. Disaster Response Core Competencies

To measure disaster response core competencies, the PCS-DMHW, developed by Yoon and Choi [21], was used. This tool measures the competencies required for mental health personnel to effectively respond to disasters. It includes 24 questions: six on knowledge (disaster understanding and customized support), nine on attitude (vocation, ethics, and qualifications), and nine on skills (problem-solving, communication, and information delivery). The answers are scored on a 5-point Likert scale ranging from 0 (not at all true) to 4 (strongly true). Total scores range from 0 to 96, with a higher score indicating a higher perception of competence in the relevant area [21]. Cronbach's *α* was 0.95 for Yoon and Choi [21] and 0.91 in this study.

#### 2.3.2. Problem-Solving Ability

Problem-solving ability was measured using the process behaviors of problem-solving developed by Lee et al. [22] and revised by Park and Woo [23]. This tool comprises 25 questions on problem discovery, problem definition, devising a solution to the problem, implementing the solution, and reviewing the solution. The answers are rated on a 5-point Likert scale ranging from 1 (not very) to 5 (almost always), with total scores ranging from 25 to 125. Higher scores indicate a better problem-solving process. Cronbach's *α* was 0.90 for Park and Woo [23] and 0.95 in this study.

#### 2.3.3. Self-Leadership Capacity

Kim [24] translated a self-leadership questionnaire developed by Manz [25] to measure self-leadership capacity. It comprises 15 questions across six subscales (self-expectation, rehearsal, goal-setting, self-reward, self-criticism, and constructive thinking). The answers are rated on a 5-point Likert scale ranging from 1 (not at all) to 5 (very much), with total scores ranging from 15 to 75. Cronbach's *α* was 0.90 for Kim [24] and 0.90 in this study.

### 2.4. Procedure

**Figure 2:**
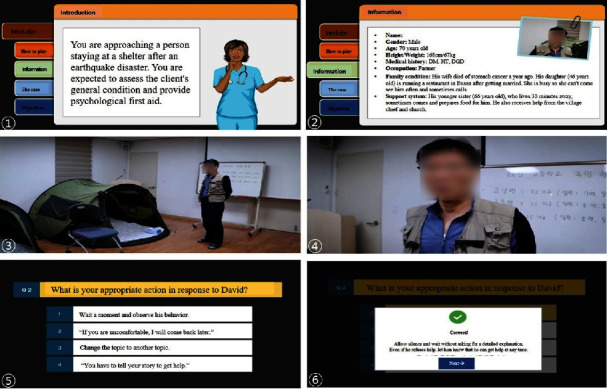
The interactive simulation game for PFA. Note: ① introducing the context of the simulation, ② providing general information of the client, ③ the first scene of the simulation, ④ interactive simulations from the learner's perspective, ⑤ taking quiz games during the simulation, and ⑥ providing real-time quiz commentaries.

The researchers created a storyboard of the pre-learning, pre-briefing, simulation, and debriefing screens. Details such as sound effects, blinking, and characters were selected for each scene, and the narration was created as an MP3 file using an artificial intelligence voice actor. The content was produced by a content development expert using Articulate Storyline 3 (Figure 2). The content was then reviewed and revised more than five times by the researcher.

A uniform resource locator (URL) allowing access to the program was sent to the experimental and control groups. By clicking on the URL, a screen opened in a new browser window, and the learner could participate in the training. The intervention was conducted between November 1 and November 7, 2020. The web-based PFA simulation applied to the experimental group was an educational program comprising four learning areas: pre-learning, pre-briefing, simulation, and debriefing (Table 1). The time required for learning ranged from 50 minutes to 1 hour. The control group was provided psychological support material in an e-book format. The e-book included only text and comprised 44 pages. After reading one page, participants had to click an arrow to move to the next page. The self-study content covered psychological support systems in the aftermath of earthquakes, characteristics of earthquakes, PFA, stabilization therapy, and frequently asked questions by earthquake victims.

### 2.5. Data Collection

Data were collected using an online questionnaire created by the researcher using Google Forms. Informed consent was obtained from all participants prior to data collection. Participants agreed to participate by checking a consent box on the first page of the Google Forms survey. The survey included items from the PCS-DMHW as well as items on problem-solving ability, critical thinking, and sociodemographic characteristics. When the researcher sent a link to the pre-questionnaire on a participant's social networking service, the participant clicked on the link and completed the questionnaire. The time required to complete the preliminary questionnaire was 15–20 minutes. Participants were required to provide their names to match the pre- and post-survey responses. The survey clearly stated that participants' names would be replaced with unique identification numbers after the completion of the post-survey. After all surveys were completed, the researcher replaced the participants' names with identification numbers. The data are stored on a password-protected computer accessible only to the researcher.

### 2.6. Data Analysis

The collected data were analyzed using SPSS/Mac SPSS 26.0. Participants' general characteristics, disaster response core competencies, problem-solving processes, and levels of self-leadership were analyzed using frequency, percentage, average, and standard deviation.

To assess pre-intervention homogeneity between the experimental and control groups, chi-square tests, Fisher's exact tests, and independent samples *t*-tests were used. Additionally, the homogeneity of the dependent variables between the experimental and control groups was assessed using independent samples *t*-tests. The normality of the distributions in both groups was tested using the Shapiro–Wilk test. Finally, to evaluate the effectiveness of the simulation education program, changes in the research variables before and after the intervention were analyzed using independent samples *t*-tests. Cohen's d was used to calculate effect sizes using means and standard deviations from pre- to post-test. According to Cohen [26], effect sizes of 0.20, 0.50, and 0.80 represent small, moderate, and large effects, respectively.

### 2.7. Ethical Considerations

This study was approved by the Chung-Ang University Institutional Review Board. Participants who voluntarily agreed to participate signed a written consent form. They were informed that they could stop or withdraw from the study at any time. Their personal information was anonymized for confidentiality. After the study, the data will be stored for three years and then disposed of in a manner that makes restoration impossible.

## 3. Results

### 3.1. Demographic and Clinical Characteristics

Table 2 presents the demographic and clinical characteristics of the participants. The mean age of the experimental group was 38.47 ± 9.37 years and that of the control group was 37.86 ± 6.85 years. The experimental and control groups had 12.83 ± 7.84 years and 12.93 ± 6.39 years of work experience, respectively. A total of 21 (70.0%) nurses in the experimental group and 23 (82.1%) in the control group had not participated in PFA training in the past; there was no statistically significant difference between both groups according to PFA training experience (*χ*^2^ = 1.17, *p*=0.363). Furthermore, there were no statistically significant differences between both groups in terms of all general characteristics; therefore, the experimental and control groups were homogeneous.

### 3.2. Effects of the Program

Table 3 presents the effects of the program. The experimental group's disaster response core competencies score increased from 51.20 ± 10.46 to 73.93 ± 12.28 after the program, while the control group's score increased from 54.68 ± 12.11 to 69.57 ± 9.44, indicating a statistically significant difference between the two groups (*t* = −2.239, *p*=0.029). This result demonstrated a medium effect size (Cohen's *d* = 0.59).

The experimental group's problem-solving process score changed from 75.90 ± 15.82 to 90.57 ± 16.56 after the program, while that of the control group increased from 76.75 ± 16.80 to 82.64 ± 17.91, indicating a statistically significant difference between the two groups (*t* = −2.753, *p*=0.008). This improvement demonstrated a medium effect size (Cohen's *d* = 0.72).

The experimental group's self-leadership score increased from 56.87 ± 9.01 to 62.07 ± 8.59 after the program, while that of the control group changed from 57.14 ± 7.72 to 59.00 ± 6.81, indicating a statistically significant difference between the two groups (*t* = −2.073, *p*=0.043). This difference demonstrated a medium effect size (Cohen's *d* = 0.54).

**Figure 3:**
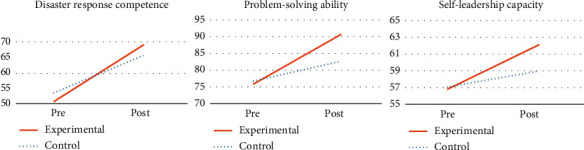
Mean differences in the study variables.

Figure 3 comprises three separate figures that illustrate the mean differences in the study variables between the experimental and control groups. From the left, the first, second, and third figures indicate disaster response core competencies, problem-solving ability, and self-leadership capacity, respectively. They highlight the changes from pre- to post-intervention for both groups.

## 4. Discussion

This study aimed to develop a web-based PFA simulation training program for nurses and verify its effectiveness. First, the results showed that the disaster response core competencies of the experimental group were significantly different from those of the control group. This result is similar to that of a study that confirmed the effectiveness of a PFA curriculum comprising three hours of theory and three hours of practice for participants, including psychiatric and nonpsychiatric experts; the participants' disaster mental health competency scores increased significantly [27]. When a large-scale disaster occurs, nurses rush to the site without being fully prepared to provide psychological support [28]. However, if nurses do not have the capacity for PFA, their confidence may decrease; they may become exhausted [27, 29] and experience secondary trauma [30]. For nurses to maintain their expertise and competency, a web-based education program that allows repetitive education and has relatively fewer time and space constraints can be helpful.

Second, the problem-solving processes of the experimental group showed a significant difference compared with that of the control group. This is consistent with a study that showed that problem-solving skills can be improved through critical thinking during PFA training [31]. Disaster situations require various problem-solving processes, but workers deployed to disaster sites lack the required critical thinking, organizational skills, and problem-solving skills and are insufficiently prepared [32]. Previous studies have shown that high-fidelity simulation education is more effective than web-based education for problem-solving processes [33]. However, the activities performed by learners who participate in web-based simulations to solve problems have a practical effect in improving problem-solving skills [34] and are recommended for education in fields where face-to-face education is difficult or dangerous. Hence, web-based training programs are needed and must be encouraged.

Third, the study results confirmed that the experimental group exhibited improved self-leadership. This finding is similar to previous studies that have reported that simulation education improves learners' self-leadership [35, 36]. Self-leadership is important for nurses at disaster sites to manage crises and work with team members and organizations [37]. A study analyzing the effects of PFA training on teamwork found that self-leadership influences safe behavior in disaster situations and helps both oneself and the organization by performing self-directed activities [38]. Although there are limited studies confirming improvement in self-leadership through web-based simulation education, which restricts direct comparisons, previous research provides some supportive evidence. For instance, a study on high-fidelity simulation education for high-risk maternity care reported improvements in students' self-leadership [35]. Another study comparing high-fidelity simulation education with video-based education found that the simulation education group demonstrated enhanced critical thinking and self-directed learning [36]. This is because simulation-based education, which includes an active problem exploration process, is more effective in improving self-leadership compared with passive education [36].

The results of this study confirmed that nurses' core disaster response competencies, problem-solving processes, and self-leadership improved after web-based PFA simulation training. They also confirm that the web-based PFA training program developed in this study can be used as educational material for nurses to provide effective PFA. Additionally, this educational program is expected to increase nurses' core disaster response capabilities in disaster situations, help them engage in effective problem-solving processes, and achieve results through a self-directed attitude.

## 5. Limitations

This study had some limitations. First, as validity was verified through convenience sampling when recruiting research participants, caution is needed when generalizing the research results. Second, the participants in this study included nurses with experience in PFA; therefore, the possibility that the effect of previous training may have influenced the results of this study cannot be ruled out. Third, a single researcher applied and evaluated the intervention, which may have introduced potential biases. Finally, the study's findings may not be widely applicable to all nurses or other healthcare professionals, as the sample was limited to a specific group. Future research should include a more diverse sample to improve the generalizability of the results.

## 6. Conclusions

This study developed a PFA training program in the form of web-based simulations. The training program reflects the work characteristics of nurses, thereby allowing learners to participate regardless of time and place and enabling repetitive education. The program provides educational opportunities for nurses to become more confident when providing psychological support to disaster-affected individuals. Nurses are expected to ultimately be trained as key practitioners who not only provide disaster psychological support but also contribute to improving the capacity for it.

## 7. Implication for Nurses' Management

The educational program developed in this study has the advantage of enabling repeated education for nurses regardless of time and place. Compared with existing PFA training, theory and practical training may be possible in a relatively shorter period of time, thus motivating learning and meeting the training needs of nurses. This increase in educational opportunities can increase nurses' confidence in providing psychological support to disaster victims. Thus, nurses are expected to ultimately be trained as key practitioners in and contribute to improving the capacity for disaster psychological support.

## Figures and Tables

**Table 1:** Content of the web-based simulation program for psychological first aid training.

Main menu	Middle menu	Submenu
Pre-learning	Earthquake	(i) Earthquake video (ii) Characteristics of earthquakes
	Disaster mental health	(i) Definition of disaster (ii) Characteristics of disaster victims
	Psychological first aid	(i) Definition of PFA (ii) Steps, preparations, attitude (iii) Basic goals (iv) Caution (v) Core activities of PFA (vi) Self-care
	Organizational structure	(vii) Role of organizations

Pre-briefing	Introduction	Introducing the simulation scenario (informing users that they must visit a disaster site and perform PFA), explaining that a quiz is presented
	How to participate	Instructions on how to participate in the quiz
	Client information	Providing general information of the client
	Client situation	Description of the subject's situation when an earthquake occurs
	Learning objectives	Present five learning goals

Simulation	12 video clips and 11 quiz games	

Debriefing	Self-debriefing—click the menu to go to the debriefing screen and write	

**Table 2:** General characteristics and homogeneity of participants (*N* = 58).

Characteristics		EG (*n* = 30)		CG (*n* = 28)		*t*/*χ*^2^	*p*
		Mean ± SD					
Years working		12.83 ± 7.84		12.93 ± 6.39		0.05	0.960

Age		38.47 ± 9.37		37.86 ± 6.85		−2.28	0.780

Frequency (%)							

Sex	Female	30	(100.0)	28	(100.0)	—	—

Religious activities	Yes	19	(63.3)	15	(53.6)	0.57	0.595
	No	11	(36.7)	11	(46.4)		

Work department	Mental health	19	(63.3)	17	(60.7)	0.04	1.000
	Others	11	(36.7)	11	(39.3)		

PFA education	Have	9	(30.0)	5	(17.9)	1.17	0.363
	None	21	(70.0)	23	(82.1)		

*Note*. CG = control group; EG = experimental group; *n* = number; SD = standard deviation.

**Table 3:** Effects of the program (*N* = 58).

Variables	Groups	Pre-test	Post-test	Difference	ES (*d*)	*t*	*p*
		Mean ± SD					
Core competencies	EG (*n* = 30)	51.20 ± 10.46	73.93 ± 12.28	22.73 ± 13.25	0.59	−2.239	0.029^∗^
	CG (*n* = 28)	54.68 ± 12.11	69.57 ± 9.44	14.89 ± 13.41			

Problem-solving	EG (*n* = 30)	75.90 ± 15.82	90.57 ± 16.56	14.67 ± 10.53	0.72	−2.753	0.008^∗∗^
	CG (*n* = 28)	76.75 ± 16.80	82.64 ± 17.91	5.89 ± 13.64			

Self-leadership	EG (*n* = 30)	56.87 ± 9.01	62.07 ± 8.59	5.20 ± 6.09	0.54	−2.073	0.043^∗^
	CG (*n* = 28)	57.14 ± 7.72	59.00 ± 6.81	1.86 ± 6.18			

*Note.* CG = control group; *d* = Cohen's *d*; EG = experimental group; ES = effect size; SD = standard deviation; ^∗^*p* < 0.05; ^∗∗^*p* < 0.01.

## Data Availability

The data of this study are available from the corresponding author upon reasonable request.
